# Radiomics Analysis of Multiparametric MRI for Prediction of Synchronous Lung Metastases in Osteosarcoma

**DOI:** 10.3389/fonc.2022.802234

**Published:** 2022-02-22

**Authors:** Zhendong Luo, Jing Li, YuTing Liao, RengYi Liu, Xinping Shen, Weiguo Chen

**Affiliations:** ^1^Department of Radiology, The University of Hong Kong - Shenzhen Hospital, Shenzhen, China; ^2^Department of Radiology, State Key Laboratory of Oncology in South China, Collaborative Innovation Center for Cancer Medicine, Sun Yat-sen University Cancer Center, Guangzhou, China; ^3^Department of Pharmaceuticals Diagnosis, GE Healthcare, Shanghai, China; ^4^Department of Radiology, Nanfang Hospital, Southern Medical University, Guangzhou, China

**Keywords:** radiomics, predictive value of tests, magnetic resonance imaging, osteosarcoma, metastasis

## Abstract

**Purpose:**

To establish and verify a predictive model involving multiparameter MRI and clinical manifestations for predicting synchronous lung metastases (SLM) in osteosarcoma.

**Materials and Methods:**

Seventy-eight consecutive patients with osteosarcoma (training dataset, n = 54; validation dataset, n = 24) were enrolled in our study. MRI features were extracted from the T1‐weighted image (T1WI), T2‐weighted image (T2WI), and contrast-enhanced T1-weighted image (CE-T1WI) of each patient. Least absolute shrinkage and selection operator (LASSO) regression and multifactor logistic regression were performed to select key features and build radiomics models in conjunction with logistic regression (LR) and support vector machine (SVM) classifiers. Eight individual models based on T1WI, T2WI, CE-T1WI, T1WI+T2WI, T1WI+CE-T1WI, T2WI+CE-T1WI, T1WI+T2WI+CE-T1WI, and clinical features, as well as two combined models, were built. The area under the receiver operating characteristic curve (AUC), sensitivity and specificity were employed to assess the different models.

**Results:**

Tumor size was the most significant univariate clinical indicator (1). The AUC values of the LR predictive model based on T1WI, T2WI, CE-T1WI, T1WI+T2WI, T1WI+CE-T1WI, T2WI+CE-T1WI, and T1WI+T2WI+CE-T1WI were 0.686, 0.85, 0.87, 0.879, 0.736, 0.85, and 0.914, respectively (2). The AUC values of the SVM predictive model based on T1WI, T2WI, CE-T1WI, T1WI+T2WI, T1WI +CE-T1WI, T2WI +CE-T1WI, and T1WI+T2WI+CE-T1WI were 0.629, 0.829, 0.771, 0.879, 0.643, 0.829, and 0.929, respectively (3). The AUC values of the clinical, combined 1 (clinical and LR-radiomics) and combined 2 (clinical and SVM-radiomics) predictive models were 0.779, 0.957, and 0.943, respectively.

**Conclusion:**

The combined model exhibited good performance in predicting osteosarcoma SLM and may be helpful in clinical decision-making.

## Introduction

Osteosarcoma is a highly prevalent primary bone malignancy. Fortunately, complete ablation of nonmetastatic high-grade osteosarcoma is possible in 60–70% of cases when treated with adjuvant and neoadjuvant multiagent chemotherapies in addition to surgery (1). However, the prognoses of osteosarcoma patients with distant metastasis remain poor. Among all forms of metastasis, lung metastasis is the most common, occurring in over 80% of patients. Approximately 20% of osteosarcoma patients also exhibit metastasis at initial diagnosis (synchronous metastases) (2, 3). The primary tumor is more resistant to chemotherapy in patients with synchronous metastases than in patients with localized disease at presentation (4). Both the number of nodules and lobes are strong indicators of survival (5). At present, the best indicators of survival are tumor grade, tumor size, and distal metastases, which can be detected from biopsies and microscopic evaluations (6). Predicting individual and early metastases is essential to osteosarcoma management, as it informs treatment strategies and increases survival rates. Chest computerized tomography (CT) has been the most commonly used imaging modality for the detection of lung nodules. Although there have been great advancements in imaging technology, particularly in enhancing the sensitivity of detection, the specificity of the data remains insufficient. Metastases cannot be properly distinguished from benign tissue (5). When nodules are detected at diagnosis, it is usually assumed that these nodules represent metastatic disease. However, not all pulmonary nodules that develop during tumor therapy are malignant, which poses additional challenges for physicians. Hence, the goal of this study was to evaluate the diagnostic abilities of two distinct classifiers (logistic regression (LR) and support vector machine (SVM)) and radiomics features retrieved from different magnetic resonance imaging (MRI) parameters, including T1-weighted imaging (T1WI), T2-weighted imaging (T2WI) and contrast-enhanced T1-weighted imaging (CE-T1WI), and the combinations of two and three of these parameters. We also developed and validated combined models according to multiparametric MRI and clinical features to predict synchronous lung metastases (SLM) in osteosarcoma.

## Materials And Methods

Our retrospective investigation was approved by the Institutional Review Board. Participant informed consent was waived due to the retrospective nature of the study.

### Patient Selection

**Figure 1:**
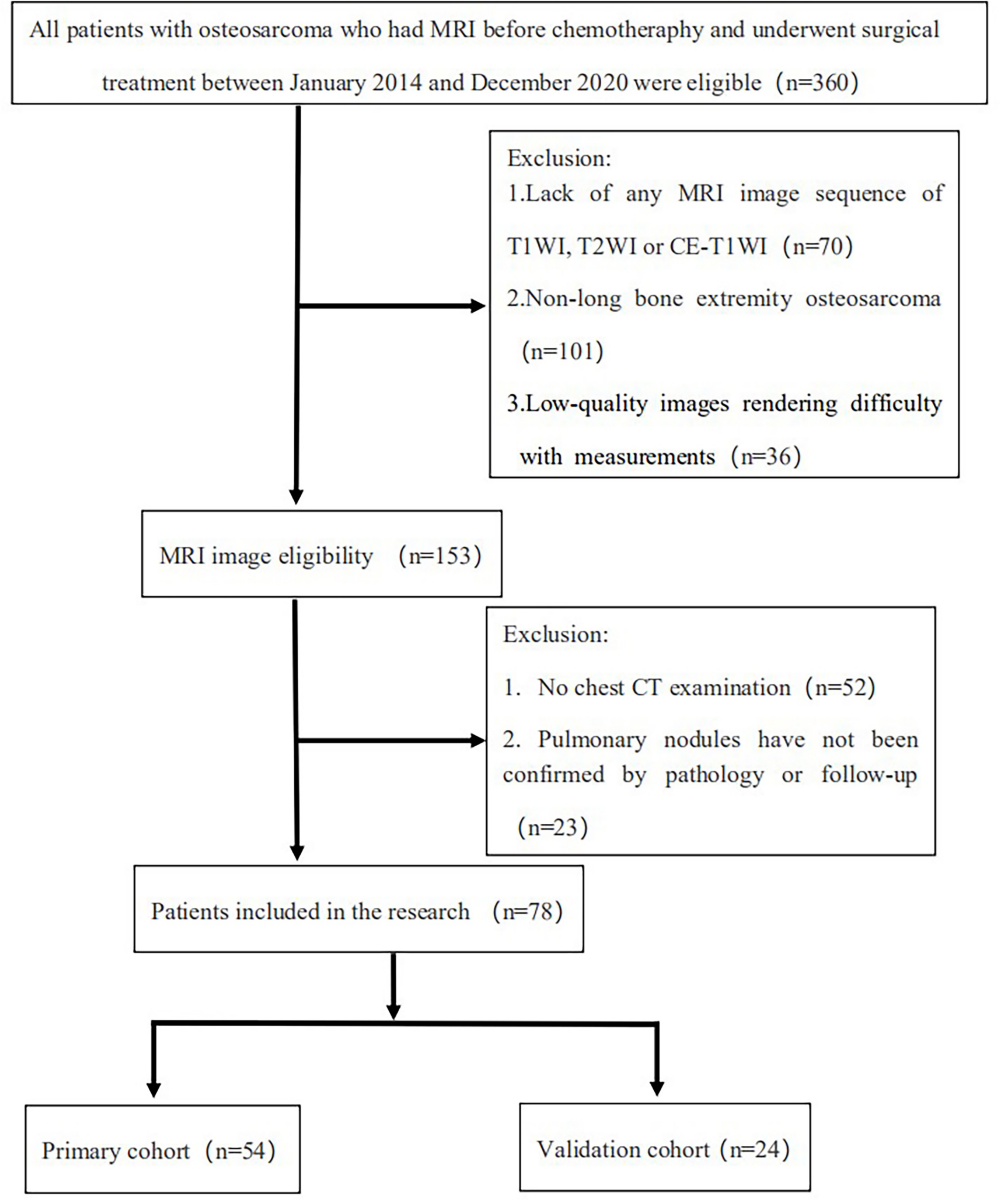
Flow chart of the study population with exclusion criteria.

Overall, 360 patients who received MRI evaluations between January 1, 2014, and December 30, 2020, were recruited for this study. The following patients were included in the study: (i) patients with no history of surgical or medical treatment administered for suspected osteosarcoma; (ii) patients who underwent multiparametric MRI, including T1WI, T2WI, and CE-T1WI, prior to treatment; (iii) patients with a osteosarcoma diagnosis confirmed by surgical resection or CT/ultrasound-guided needle biopsy and histopathological results; and (iv) patients diagnosed with SLM according to follow-up chest CT or confirmed by pathology. The patients in this study had lung nodules, and the possibility of viral, bacterial or fungal infection was ruled out. Three criteria were used to identify SLM lung nodules on follow-up chest CT according to previous studies (7, 8): first, the presence of multiple round nodules with or without changes in size or number; second, nodule size ≥5 mm and the presence of calcifications or ossification that remained stable or increased in size relative to the initial chest CT; third, changes in size or morphology during chemotherapy. Fifteen and 18 patients with SLM were diagnosed by biopsy and follow-up chest CT, respectively. The following patients were excluded from this study: (i) patients who received biopsy and locoregional therapy before MRI; (ii) patients with low-quality images rendering analysis difficult (such as images with metallic artefacts or motion artefacts); and (iii) patients with missing images or relevant sequences. A schematic diagram of our patient selection process is provided in **Figure 1**. After the application of these criteria, 78 patients were eligible for this study. The clinical characteristics of the 78 osteosarcoma patients divided into non-SLM and SLM groups are shown in **Table 1**. We next arbitrarily divided the patients into two populations: 54 patients were placed in the training cohort (TC) and 24 were placed in the validation cohort (VC) based on the seed point set obtained from programming. The clinical characteristics of the 78 osteosarcoma patients in the TC and VC are shown in **Table 2**, and a further breakdown of the clinical characteristics of these cohorts in terms of SLM and lack of SLM of osteosarcoma are summarized in **Table 3**.

**Table 1:** Clinical characteristics of 78 cases of osteosarcoma.

Characteristic	Non-SLM	SLM	*P *value
Sex			0.412
Female	15 (33.33%)	14 (42.42%)	
Male	30 (66.67%)	19 (57.58%)	
Pathology			0.984
Osteoblastic	34 (75.56%)	25 (75.76%)	
Others	11 (24.44%)	8 (24.24%)	
Location			0.486
Femur	29 (64.44%)	17 (51.52%)	
Tibia	8 (17.78%)	7 (21.21%)	
Others	8 (17.78%)	9 (27.27%)	
Bone destruction			0.067
Mix	21 (46.67%)	14 (42.42%)	
Osteolytic	22 (48.89%)	12 (36.36%)	
Osteoblastic	2 (4.44%)	7 (21.21%)	
Age (years)	19.49 ± 13.86	16.45 ± 7.53	0.258
Tumor size (cm)	6.31 ± 1.32	8.09 ± 2.39	<0.001*
ALP (IU/L)	758.49 ± 2286.19	913.30 ± 1659.41	0.742
LDH (IU/L)	256.81 ± 105.03	347.63 ± 312.71	0.073

SLM, synchronous lung metastases; ALP, alkaline phosphatase; LDH, lactate dehydrogenase.

*p < 0.05.

**Table 2:** The clinical characteristics of the 78 osteosarcoma patients in the training and validation cohorts.

Characteristic	Training cohorts	Validation cohorts	*P *value
Sex			0.056
Female	24 (44.44%)	5 (20.83%)	
Male	30 (55.56%)	19 (79.17%)	
Pathology			0.291
Osteoblastic	39 (72.22%)	20 (83.33%)	
Others	15 (27.78%)	4 (16.67%)	
Location			0.322
Femur	34 (62.96%)	12 (50.00%)	
Tibia	8 (14.81%)	7 (29.17%)	
Others	12 (22.22%)	5 (20.83%)	
Bone destruction			0.216
Mix	27 (50.00%)	8 (33.33%)	
Osteolytic	20 (37.04%)	14 (58.33%)	
Osteoblastic	7 (12.96%)	2 (8.33%)	
Age (years)	16.52 ± 9.49	22.00 ± 15.00	0.109
Tumor size (cm)	7.25 ± 1.95	6.66 ± 2.19	0.241
ALP (IU/L)	679.24 ± 1335.34	1149.65 ± 3095.32	0.349
LDH (IU/L)	303.17 ± 250.44	277.36 ± 137.72	0.638

ALP, alkaline phosphatase; LDH, lactate dehydrogenase.

**Table 3:** The clinical characteristics of these cohorts in terms of SLM and non-SLM of osteosarcoma.

Characteristic	Training cohorts		*P*	Validation cohorts		*P*
	Non-SLM	SLM		Non-SLM	SLM	
Sex			0.667			0.615
Female	13 (41.94%)	11 (47.83%)		2 (14.29%)	3 (30.00%)	
Male	18 (58.06%)	12 (52.17%)		12 (85.71%)	7 (70.00%)	
Pathology			0.707			0.615
Osteoblastic	23 (74.19%)	16 (69.57%)		11 (78.57%)	9 (90.00%)	
Others	8 (25.81%)	7 (30.43%)		3 (21.43%)	1 (10.00%)	
Location			0.169			0.202
Femur	21 (67.74%)	13 (56.52%)		8 (57.14%)	4 (40.00%)	
Tibia	6 (19.35%)	2 (8.70%)		2 (14.29%)	5 (50.00%)	
Others	4 (12.90%)	8 (34.78%)		4 (28.57%)	1 (10.00%)	
Bone destruction			0.261			0.125
Mix	17 (54.84%)	10 (43.48%)		4 (28.57%)	4 (40.00%)	
Osteolytic	12 (38.71%)	8 (34.78%)		10 (71.43%)	4 (40.00%)	
Osteoblastic	2 (6.45%)	5 (21.74%)		0 (0.00%)	2 (20.00%)	
Age (years)	15.81 ± 10.49	17.48 ± 8.07	0.527	27.64 ± 17.10	14.10 ± 5.78	0.014*
Tumor size (cm)	6.47 ± 1.38	8.29 ± 2.14	0.001*	5.96 ± 1.12	7.64 ± 2.94	0.113
ALP (IU/L)	460.17 ± 455.98	974.52 ± 1963.01	0.164	1419.05 ± 4065.50	772.50 ± 582.77	0.625
LDH (IU/L)	256.58 ± 83.78	365.97 ± 366.54	0.173	257.30 ± 145.39	305.44 ± 128.21	0.411

SLM, synchronous lung metastases; ALP, alkaline phosphatase; LDH, lactate dehydrogenase. *p < 0.05.

Patient clinical features, such as age, sex, tumor size, pathological type, tumor location, bone destruction type, and alkaline phosphatase (ALP) and lactate dehydrogenase (LDH) levels, were recorded.

### MR Imaging

All MR imaging was conducted with 1.5- or 3.0-T superconducting magnet systems. The imaging sequences included axial T1WI, T2WI and CE-T1WI. The detailed scan parameters of the four MRI scanners are described in **Table 4**. Gadolinium contrast agent was intravenously administered *via* a weight-based dosing protocol (0.1 mmol/kg) at an injection rate of 2.5 mL/s. All the MR data were obtained from the picture archiving and communication system (PACS) of our institutes and stored in Digital Imaging and Communications in Medicine (DICOM) format for additional analyses.

**Table 4:** The detailed scan parameters of four MRI scanners.

Sequence	Imaging planes	Category	TR (ms)	TE (ms)	FOV (mm×mm)	Matrix	Intersection gap (mm)	Slice thickness (mm)
T1WI	Axial	FSE	457-709	8.4-13.2	180×180~ 380×380	320×128~ 448×257	0	3-6
T2WI	Axial	FSE	3,640-7,904	83-95.2	180×180~ 380×380	320×128~ 448×257	0	3-6
CE-T1WI	Axial	FSE	457-709	8.4-13.2	180×180~ 380×380	320×128~ 448×257	0	3-6

MRI, magnetic resonance imaging; TR, repetition time; TE, echo time; FOV, field of view; T1WI, T1-weighted imaging; T2WI, T2-weighted imaging; FSE, fast spin echo; CE, contrast-enhanced.

### Preprocessing of MR Images

**Figure 2:**
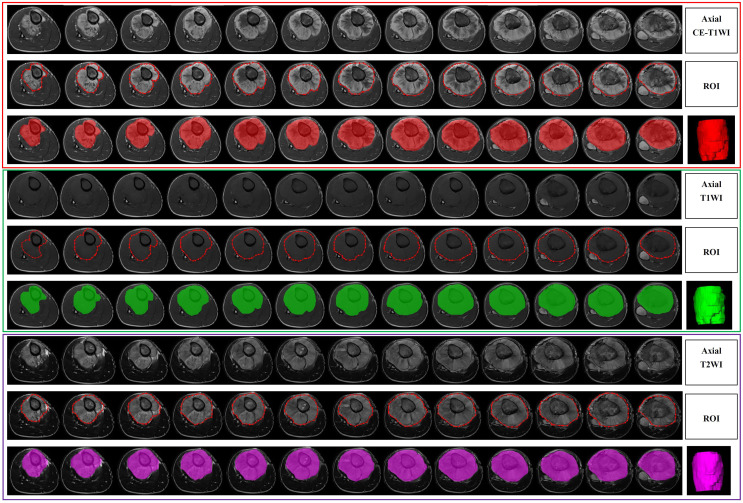
An example of a segmented MRI image.

All the images were exported to ITK-SNAP software (version 3.8.0, http://www.itksnap.org/) for segmentation before radiomics analysis. Lesion segmentation was performed by a radiologist with over 5 years of MRI diagnostic experience, and proper segmentation was further confirmed by a separate radiologist with over 10 years of MRI diagnostic experience. If disagreements arose about a specific image segmentation, a revision was made by two radiologists after discussion. The segmentation for 24 randomly selected patients was then repeated by another radiologist (over 10 years of experience). A separate region of interest (ROI) was manually selected for all sequences on each axial T1WI, T2WI and CE-T1WI slice. All the images were acquired without fat suppression. Delineation of the ROI, including the entire tumor and necrotic areas, cyst degeneration, hemorrhage, periosteal reactions, and peritumoral oedema, was carried out on the images from each sequence. **Figure 2** shows an example of a segmented MRI image. Image intensity normalization was performed before feature extraction, including image gray normalization to uniform grayscale of 0‐255 and resampling to 1 mm ×1 mm× 1 mm voxel size using linear interpolation by AK software (Analysis Kit; GE Healthcare).

### Radiomic Feature Extraction

In total, 944 radiomic features, quantifying phenotypic differences on the basis of shape (reflecting the size and shape of tumors), first-order (measuring the signal intensity of different tumors), and texture features (representing the relationship between each tumor voxel and its surrounding environments) (9), were automatically extracted from each segmented region of interest by using in-house software written in Python (Pyradiomics version: V 3.0; https://github.com/Radiomics/pyradiomics) (10). All the features were calculated in 3D directions within the whole-tumor volume and normalized by transforming the data into standardized intensity ranges (z-score transformation). Intraclass correlation coefficients (ICCs) based on a multiple-rating, consistency, 2-way random-effects model were calculated to assess the stability and reproducibility of radiomic features. For both tumor ROIs, only features with an ICC > 0.75 were considered to suggest good agreement and retained for further radiomic feature selection.

### Radiomics Feature Selection

Radiomics features were automatically calculated with the noncommercial Analysis Kit (A.K. GE Healthcare). First, we performed least absolute shrinkage and selection operator (LASSO) regression on all features to grossly choose attributes with discriminative ability. The goal was to reduce certain attribute coefficients to zero by regulating parameter λ. Subsequently, the area under the receiver operating characteristic (ROC) curve (AUC) could be determined versus log(λ) by employing tenfold cross-validation. The advantage of the LASSO technique is that it can analyse a massive amount of radiomics characteristics from low numbers of samples. Second, we applied multivariate logistic regression to select the most predictive features.

### Machine Learning Model

This study used two machine learning classifiers: LR and SVM.

An SVM model was generated based on the established optimal feature subsets of the TC dataset. The kernel, gamma, degree, coef, and C parameters were set to ‘rbf’, 0.0, 3, 0.0, and 1.0, respectively.

The individual sequence models were constructed by T1WI, T2WI and CE-T1WI.

Next, four combined models were generated *via* a combination of features of dissimilar sequences, namely, T1WI+T2WI, T1WI+CE-T1WI, T2WI+CE-T1WI, and T1WI+T2WI+CE-T1WI. Clinical features were analysed by univariate analysis, and variables for which P < 0.05 were entered into the clinical model.

Two combined models were constructed by combining the best LR and SVM radiomics models with clinical features.

The models were conditioned with the TC using the repeated 10-fold cross-validation technique, and their performance was assessed in the VC.

**Figure 3:**
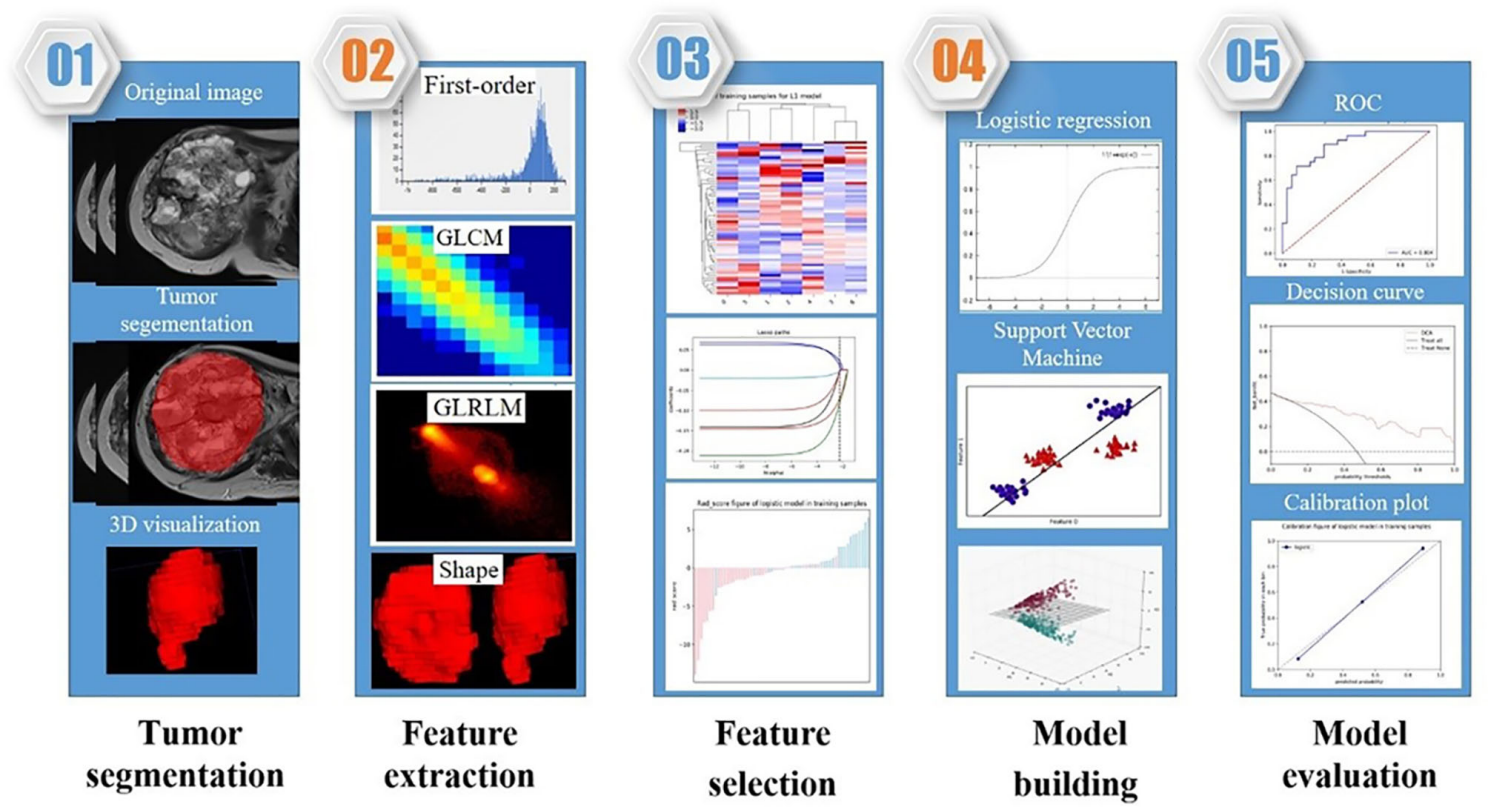
The radiomics framework of our study.

The radiomics framework of our study is shown in **Figure 3**.

### Statistical Analysis

The t-test or Mann-Whitney U-test was employed for the comparison of continuous variables, whereas the chi-squared test or Fisher’s exact test was employed for the comparison of intergroup categorical variables. All the statistical analyses were two-sided, and a Bonferroni-corrected P value was employed to determine the feature significance of multiple comparisons. ROC curves were generated to assess the performance of the machine learning models, and the sensitivity, specificity and AUC values were calculated. The AUCs of each two models (clinical, radiomics and combined models) in the two cohorts were compared by using the DeLong test. All the data analyses were performed in R 3.5.1 and Python 3.5.6. A two-tailed P value <0.05 was set as the significance threshold.

## Results

### Clinical Characteristics of the Patients

In total, 78 osteosarcoma patients (49 males, 29 females; between 15-83 years of age) were recruited. Based on our univariate analysis, the tumor size was markedly different between the two groups (P < 0.05) (**Table 1**). No obvious differences were observed in age, sex, pathological type, tumor location, bone destruction type, and ALP or LDH levels between the SLM and non-SLM groups. Moreover, no marked differences were observed between the TC and VC (**Table 2**). In addition, the clinical features were not markedly different between the SLM and non-SLM cohorts, except for age in the VC and tumor size in the TC (**Table 3**).

### Performance of the Radiomics Models

Overall, 944 radiomics features were obtained from each of the T1WI, T2WI and CE-T1WI images. A total of 702, 839 and 835 radiomics features from T1WI, T2WI and CE-T1WI were included, respectively, with ICC greater than 0.75. The radiomics features with the largest differences between the models are summarized in **Table 5**.

**Table 5:** The most significant radiomics features of different models.

Model	Radiomics features	Coef.
T1WI	Intercept	-0.4597
	T1WI_wavelet-LLL_glcm_Correlation	1.3060
	T1WI_wavelet-LLL_gldm_GrayLevelNonUniformity	0.8114
T2WI	Intercept	-1.6745
	T2WI_wavelet-LLL_glcm_Correlation	3.4374
	T2WI_wavelet-HHH_firstorder_Mean	-2.7065
	T2WI_wavelet-HLH_glcm_MCC	-3.5169
	T2WI_wavelet-LHL_gldm_LargeDependenceHighGrayLevelEmphasis	2.1317
CE-T1WI	Intercept	-1.2416
	CE-T1WI_wavelet-LLL_glcm_Correlation	1.6253
	CE-T1WI_wavelet-LHL_firstorder_Mean	-1.4719
	CE-T1WI_wavelet-HHL_firstorder_Skewness	-1.7320
	CE-T1WI_wavelet-HLH_glcm_MCC	-1.3359
	CE-T1WI_wavelet-LHH_firstorder_Kurtosis	1.1668
T1WI+T2WI	Intercept	-1.0635
	T2WI_wavelet-LLL_glcm_Correlation	2.2998
	T2WI_wavelet-HHH_firstorder_Mean	-1.5630
	T2WI_log-sigma-1-0-mm-3D_ngtdm_Busyness	1.1750
	T2WI_wavelet-HHH_gldm_SmallDependenceLowGrayLevelEmphasis	-1.2490
T1WI+CE-T1WI	Intercept	-0.8489
	T1WI_wavelet-LLL_glcm_Correlation	1.2206
	CE-T1WI_wavelet-LHL_firstorder_Mean	-1.6295
	CE-T1WI_wavelet-HHL_firstorder_Skewness	-1.1276
T2WI+CE-T1WI	Intercept	-1.6745
	T2WI_wavelet-LLL_glcm_Correlation	3.4374
	T2WI_wavelet-HHH_firstorder_Mean	-2.7065
	T2WI_wavelet-HLH_glcm_MCC	-3.5169
	T2WI_wavelet-LHL_gldm_LargeDependenceHighGrayLevelEmphasis	2.1317
T1WI+T2WI+CE-T1WI	Intercept	-1.2077
	T2WI_wavelet-LLL_glcm_Correlation	2.4347
	T2WI_wavelet-HHH_firstorder_Mean	-1.6936
	CE-T1WI_wavelet-HLH_glcm_MCC	-1.5491

**Figure 4:**
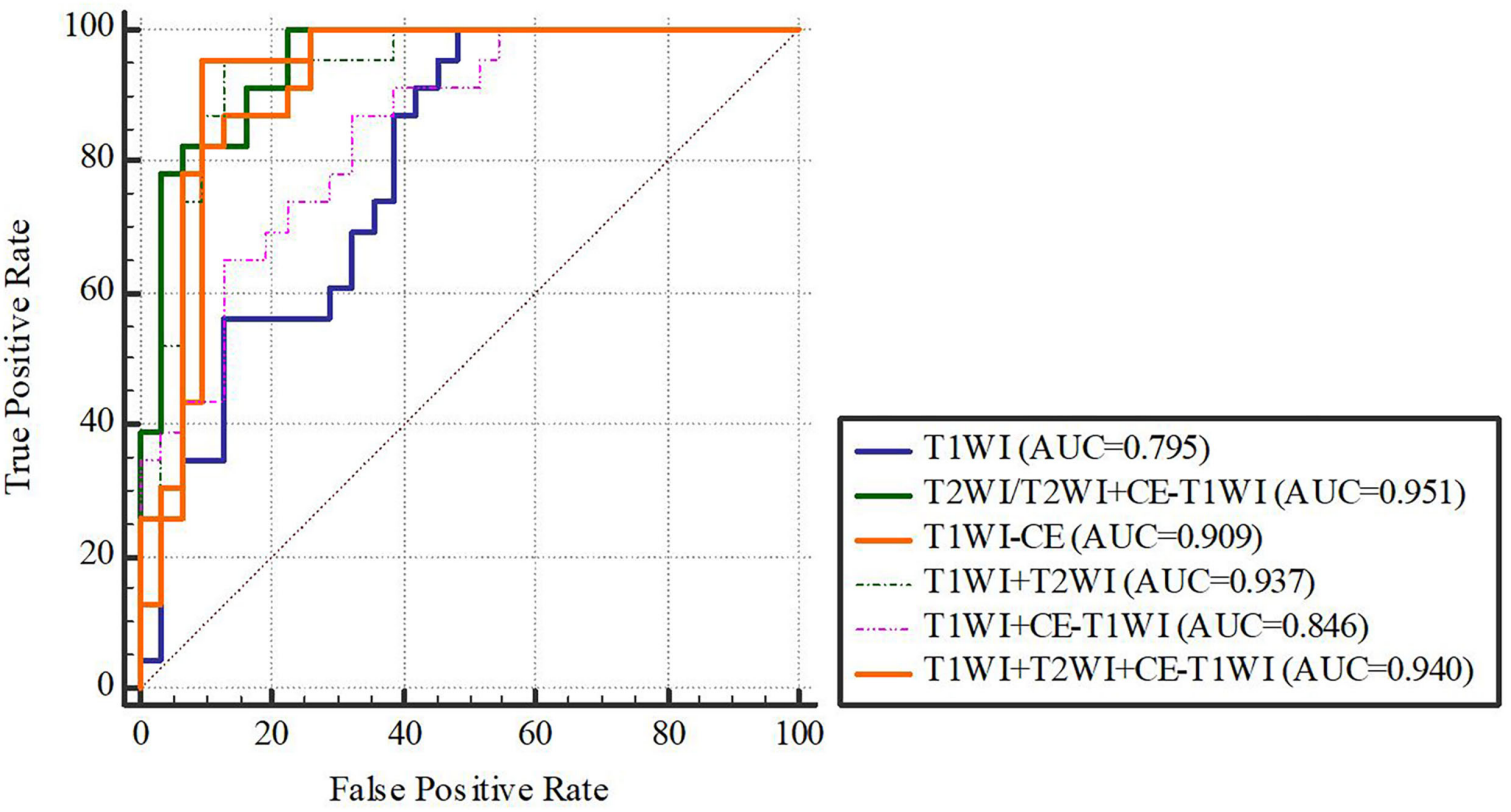
LR-classifier in the training cohort.

**Figure 5:**
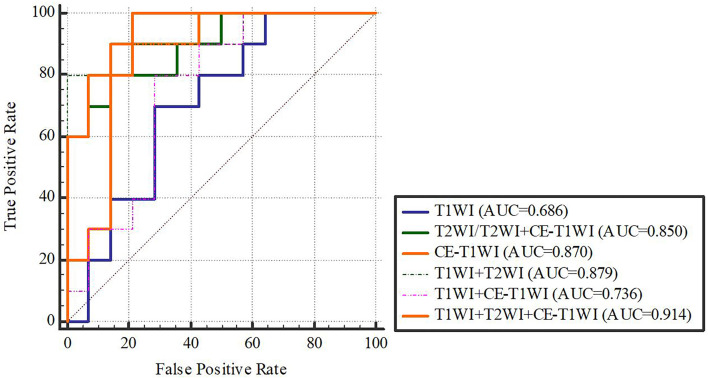
LR-classifier in the validation cohort.

In terms of a distinct sequence in the LR classifier, CE-T1WI features displayed a stronger predictive performance (AUC = 0.87, 95% CI, 0.655-0.965) than T2WI (AUC = 0.85, 95% CI, 0.699-0.981) and T1WI (AUC = 0.686, 95% CI, 0.488-0.873) features in the VC. In terms of combined features, T1WI+T2WI+CE-T1WI had a higher performance (AUC = 0.914, 95% CI, 0.776-0.998) than T1WI+CE-T1WI (AUC = 0.736, 95% CI, 0.533-0.902), T2WI+CE-T1WI (AUC = 0.85, 95% CI, 0.699-0.981) and T1WI+T2WI (AUC = 0.879, 95% CI, 0.746-0.993) (**Tables 6**, **7** and **Figures 4**, **5**).

**Table 6:** The ROC curve of different models of LR-classifier in the training cohort.

Classifiers	Model	AUC	95% CI	Sensitivity	Specificity
LR	T1WI	0.795	0.663 - 0.893	0.565	0.806
	T2WI	0.951	0.855 - 0.991	0.826	0.864
	CE-T1WI	0.909	0.799 - 0.970	0.870f	0.871
	T1WI+T2WI	0.937	0.836 - 0.985	0.783	0.903
	T1WI+CE-T1WI	0.846	0.722 - 0.930	0.739	0.774
	T2WI+CE-T1WI	0.951	0.855 - 0.991	0.826	0.864
	T1WI+T2WI+CE-T1WI	0.940	0.840 - 0.986	0.913	0.903

T1WI, T1-weighted imaging; T2WI, T2-weighted imaging; AUC, area under curve; 95% CI, 95% confidence interval; LR, logistic regression.

**Table 7:** The ROC curve of different models of LR-classifier in the validation cohort.

Classifiers	Model	AUC	95% CI	Sensitivity	Specificity
LR	T1WI	0.686	0.488 - 0.873	0.400	0.786
	T2WI	0.850	0.699 - 0.981	0.600	0.750
	CE-T1WI	0.870	0.655 - 0.965	0.500	0.786
	T1WI+T2WI	0.879	0.746 - 0.993	0.700	0.929
	T1WI+CE-T1WI	0.736	0.533 - 0.902	0.400	0.786
	T2WI+CE-T1WI	0.850	0.699 - 0.981	0.600	0.750
	T1WI+T2WI+CE-T1WI	0.914	0.776 - 0.998	0.700	0.929

T1WI, T1-weighted imaging; T2WI, T2-weighted imaging; AUC, area under curve; 95% CI, 95% confidence interval; LR, logistic regression.

Delong-test results in **Table 8** showed that there were significant differences between predictive performance of T1WI-radiomic model and that of T1WI+T2WI+CE-T1WI radiomic model in both cohorts.

**Table 8:** Delong Test between each two models of LR-classifier in the training and validation cohorts.

Radiomic model	T1WI	T2WI	CE-T1WI	T1WI+T2WI	T1WI+CE-T1WI	T2WI+CE-T1WI	T1WI+T2WI+CE-T1WI
T1WI	–	0.0063*	0.0725	0.0117*	0.4114	0.0063*	0.0110*
T2WI	0.1391	–	0.2395	0.6474	0.0318*	1.0000	0.6744
CE-T1WI	0.1048	0.7088	–	0.4144	0.1713	0.2395	0.3033
T1WI+T2WI	0.0695	0.5149	0.4838	–	0.0613	0.6474	0.8620
T1WI+CE-T1WI	0.6829	0.2054	0.2238	0.0771	–	0.0318*	0.0545
T2WI+CE-T1WI	0.1391	1.0000	0.7088	0.5149	0.2054	–	0.6744
T1WI+T2WI+ CE-T1WI	0.0465*	0.2542	0.3188	0.6750	0.0720	0.2542	–
Training cohort				Validation cohort			

T1WI, T1-weighted imaging; T2WI, T2-weighted imaging; CE, contrast-enhanced. *p < 0.05.

**Figure 6:**
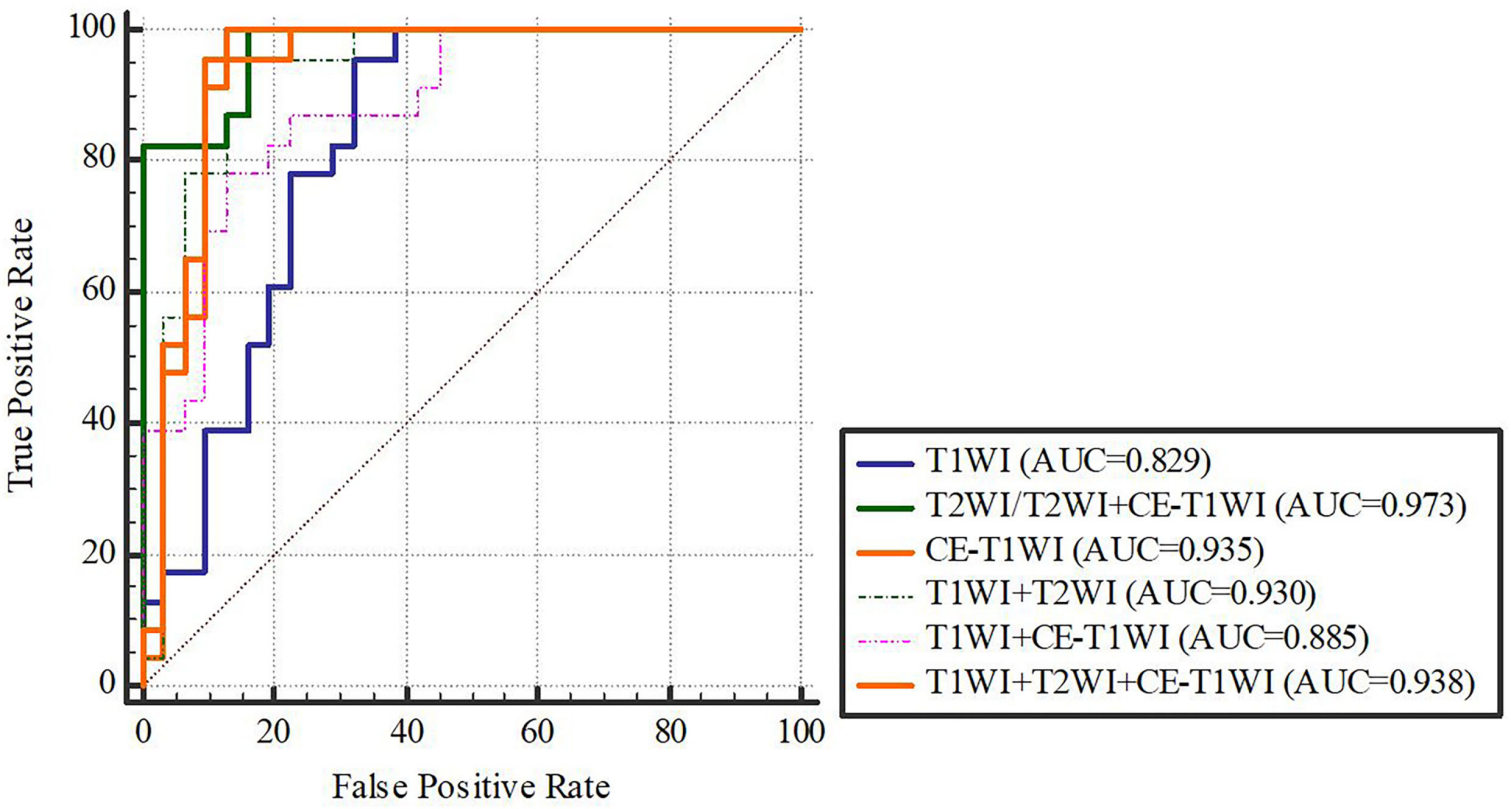
SVM-classifier in the training cohort.

**Figure 7:**
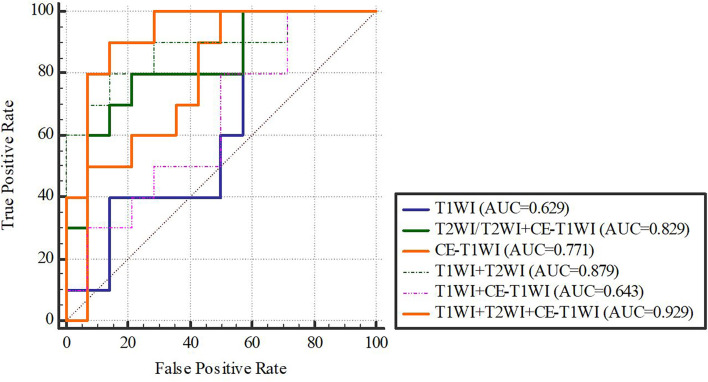
SVM-classifier in the validation cohort. **Figures 4**–**7** The ROC curve of different models and classifier in the training and validation cohorts.

In terms of a distinct sequence in the SVM classifier, T2WI features were more enhanced (AUC = 0.829, 95% CI, 0.621-0.950), compared to CE-T1WI (AUC = 0.771, 95% CI, 0.556-0.916) and T1WI (AUC = 0.629, 95% CI, 0.409-0.815) features in the VC. In terms of the combined features, T1WI+T2WI+CE-T1WI had a higher performance (AUC = 0.929, 95% CI, 0.746-0.993) than T1WI+CE-T1WI (AUC = 0.643, 95% CI, 0.423-0.826), T2WI+CE-T1WI (AUC = 0.829, 95% CI, 0.621-0.950) and T1WI+T2WI (AUC = 0.879, 95% CI, 0.681-0.975) (**Tables 9**, **10** and **Figures 6**, **7**).

**Table 9:** The ROC curve of different models of SVM-classifier in the training cohort.

Classifiers	Model	AUC	95% CI	Sensitivity	Specificity
SVM	T1WI	0.829	0.702 - 0.918	0.957	0.677
	T2WI	0.973	0.888 - 0.998	1.000	0.838
	CE-T1WI	0.935	0.834 - 0.984	1.000	0.871
	T1WI+T2WI	0.930	0.826 - 0.981	0.957	0.839
	T1WI+CE-T1WI	0.885	0.769 - 0.956	0.783	0.871
	T2WI+CE-T1WI	0.973	0.888 - 0.998	1.000	0.839
	T1WI+T2WI+CE-T1WI	0.938	0.838 - 0.986	0.957	0.903

T1WI, T1-weighted imaging; T2WI, T2-weighted imaging; AUC, area under curve; 95% CI, 95% confidence interval; SVM, support vector machine.

**Table 10:** The ROC curve of different models of SVM-classifier in the validation cohort.

Classifiers	Model	AUC	95% CI	Sensitivity	Specificity
SVM	T1WI	0.629	0.409 - 0.815	1.000	0.429
	T2WI	0.829	0.621 - 0.950	0.800	0.786
	CE-T1WI	0.771	0.556 - 0.916	1.000	0.500
	T1WI+T2WI	0.879	0.681 - 0.975	0.800	0.857
	T1WI+CE-T1WI	0.643	0.423 - 0.826	0.800	0.500
	T2WI+CE-T1WI	0.829	0.621 - 0.950	0.800	0.786
	T1WI+T2WI+CE-T1WI	0.929	0.746 - 0.993	0.900	0.857

T1WI, T1-weighted imaging; T2WI, T2-weighted imaging; AUC, area under curve; 95% CI, 95% confidence interval; SVM, support vector machine.

Delong-test results in **Table 11** showed that there were significant differences between predictive performance of T1WI-radiomic model and that of T1WI+T2WI+CE-T1WI radiomic model in validation cohort.

**Table 11:** Delong Test between each two models of SVM-classifier in the training and validation cohorts.

Radiomic model	T1WI	T2WI	CE-T1WI	T1WI+T2WI	T1WI+CE-T1WI	T2WI+CE-T1WI	T1WI+T2WI+CE-T1WI
T1WI	–	0.0084*	0.1247	0.1305	0.2795	0.0084*	0.0956
T2WI	0.0928	–	0.2589	0.1822	0.0449*	1.0000	0.2545
CE-T1WI	0.2519	0.4948	–	0.8594	0.3360	0.2589	0.9250
T1WI+T2WI	0.0306*	0.4472	0.2551	–	0.3718	0.1822	0.5620
T1WI+CE-T1WI	0.8981	0.1217	0.1746	0.0203*	–	0.0449*	0.2863
T2WI+CE-T1WI	0.0928	1.0000	0.4948	0.4472	0.1217	–	0.2545
T1WI+T2WI+ CE-T1WI	0.0079*	0.1404	0.0912	0.3870	0.0125*	0.1404	–
Training cohort				Validation cohort			

T1WI, T1-weighted imaging; T2WI, T2-weighted imaging; CE, contrast-enhanced. *p < 0.05.

**Figure 8:**
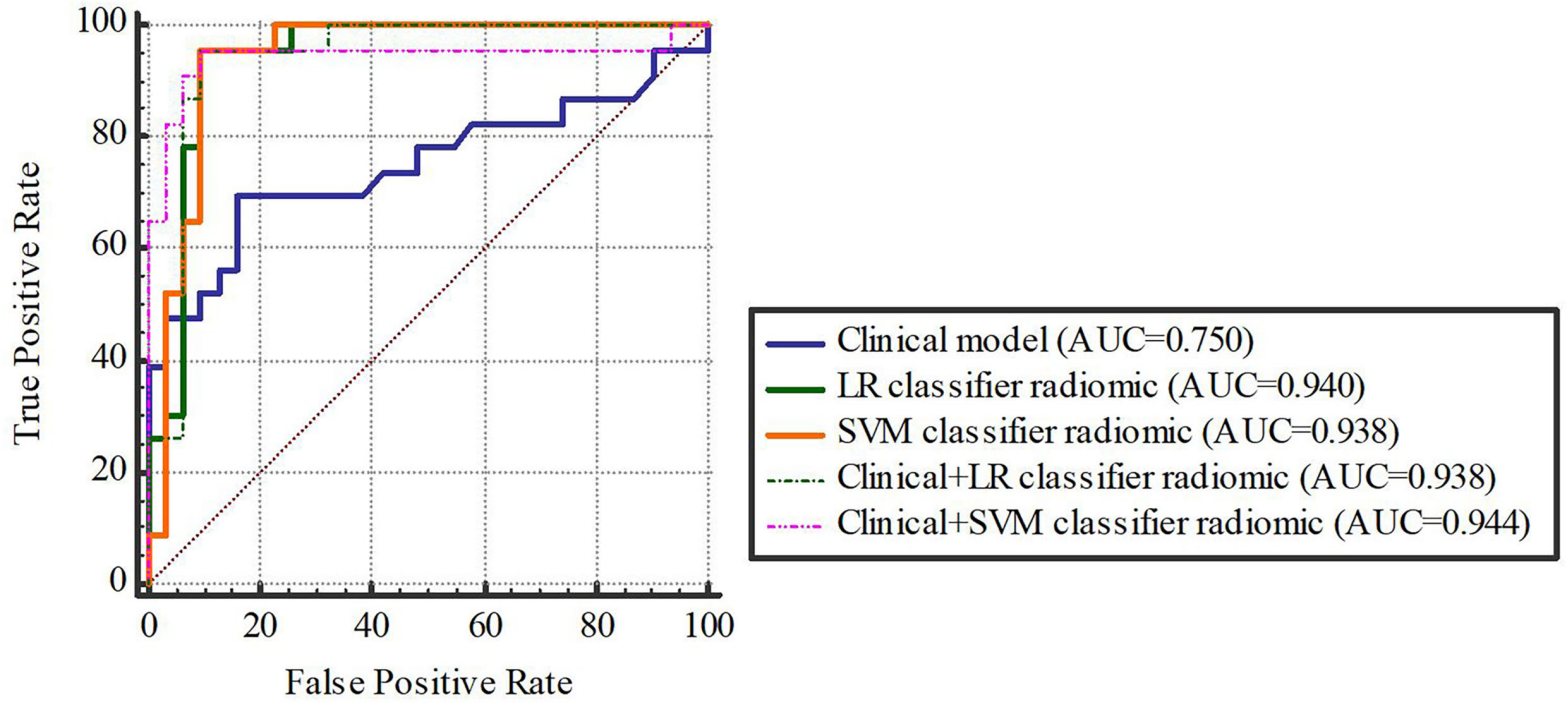
The training cohort.

**Figure 9:**
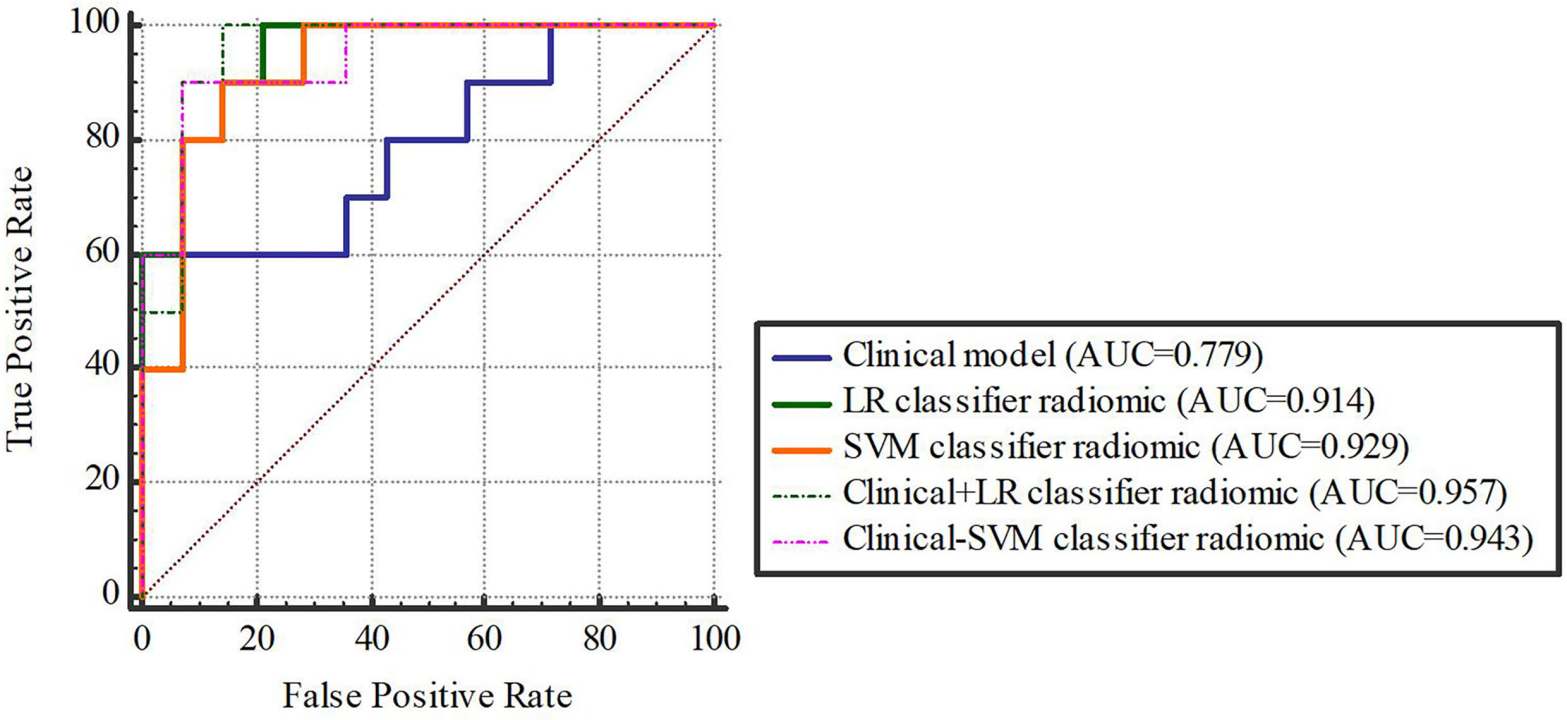
The validation cohort. **Figures 8**, **9** The ROC curve of clinical features, radiomic, clinical features + radiomic model in the training and validation cohorts.

Based on our univariate analysis, marked differences were observed in tumor size between the non-SLM and SLM sets (P < 0.05). Thus, the clinical model was built using tumor size alone, and this model performed well in the TC (AUC = 0.75, 95% CI, 0.613-0.858) and VC (AUC = 0.779, 95% CI, 0.564-0.921). When tumor size was combined with radiomics features, the combined model achieved enhanced prediction compared to the clinical model. The first combined model involving LR-radiomics features + clinical features had an AUC of 0.938 (95% CI, 0.838-0.986) in the TC and 0.957 (95% CI, 0.787-0.999) in the VC. The second combined model based on SVM-radiomics features + clinical features had an AUC of 0.944 (95% CI, 0.845-0.988) in the TC and 0.943 (95% CI, 0.766-0.997) in the VC (**Tables 12**, **13** and **Figures 8**, **9**).

**Table 12:** The ROC curve of clinical features, radiomic, clinical features + radiomic model in the training cohort.

Model	AUC	95% CI	Sensitivity	Specificity
Clinical model	0.750	0.613 - 0.858	0.696	0.839
LR-radiomic	0.940	0.840 - 0.986	0.913	0.903
SVM-radiomic	0.938	0.838 - 0.986	0.957	0.903
Combined 1 (clinical+LR-radiomic)	0.938	0.838 - 0.986	0.957	0.903
Combined 2 (clinical+SVM-radiomic)	0.944	0.845 - 0.988	0.956	0.900

AUC, area under curve; LR, logistic regression; 95% CI, 95% confidence interval; SVM, support vector machine.

**Table 13:** The ROC curve of clinical features, radiomic, clinical features + radiomic model in the validation cohort.

Model	AUC	95% CI	Sensitivity	Specificity
Clinical model	0.779	0.564 - 0.921	0.600	0.929
LR-radiomic	0.914	0.776 - 0.998	0.700	0.929
SVM-radiomic	0.929	0.746 - 0.993	0.900	0.857
Combined 1 (clinical+LR-radiomic)	0.957	0.787 - 0.999	1.000	0.857
Combined 2 (clinical+SVM-radiomic)	0.943	0.766 - 0.997	0.846	0.929

AUC, area under curve; LR, logistic regression; 95% CI, 95% confidence interval; SVM, support vector machine.

Delong-test results in **Table 14** indicated that there were insignificant differences between predictive performance of combined mode and that of clinical model, LR-Radiomic model and SVM-Radiomic model in both cohorts. The combined model 1 achieved equivalent AUCs compared to combined model 2. Additionally, the combined model 1 performed better than the clinical model as indicated by AUCs of borderline statistical significance (p = 0.0619) in the validation cohort.

**Table 14:** Delong Test between each two models (clinical, radiomic, clinical features + radiomic model) in the training and validation cohort.

Model	Clinical	LR-Radiomic	SVM-Radiomic	Clinical +LR-Radiomic	Clinical +SVM-Radiomic
Clinical	–	0.0140*	0.0161*	0.0138*	0.0085*
LR-Radiomic	0.0966	–	0.9065	0.7927	0.9142
SVM-Radiomic	0.1361	0.5247	–	1.0000	0.8908
Combined 1 (Clinical +LR-Radiomic)	0.0619	0.7807	0.2536	–	0.8798
Combined 2 (Clinical +SVM-Radiomic)	0.0787	0.8381	0.6251	0.6265	–
Training cohort			Validation cohort		

T1WI, T1-weighted imaging; T2WI, T2-weighted imaging; CE, contrast-enhanced; LR, logistic regression; SVM, support vector machine. *p < 0.05.

## Discussion

According to our univariate analysis, tumor size was the most reliable indicator of SLM in osteosarcoma patients, which is in accordance with prior findings (11–14). Huang et al (11) performed a retrospective study examining the characteristics of 1057 osteosarcoma patients. These authors reported that large tumors (>5 cm) were at a substantially elevated risk of resulting in lung metastases in osteosarcoma patients. Munajat et al. (13) also examined the correlation between lung metastasis and tumor volume in a population of 70 osteosarcoma patients. These authors reported that 33 patients (47%), who mostly exhibited larger tumor volumes, also showed signs of lung metastasis. However, in contrast to our study, these authors primarily concentrated on lung metastasis without distinguishing synchronous and metachronous metastases. In our study, we compared tumor size (in terms of diameter) between the non-SLM (6.31 ± 1.32 cm) and SLM (8.09 ± 2.39 cm) groups and found that the tumor size in the SLM group was significantly larger than that in the non-SLM group.

Tumor heterogeneity strongly modulates tumor invasion and prognosis, and a radiomics profile can specifically reflect the complicated histopathology of tumor (15, 16). Several MRI radiomics studies conducted on osteosarcoma were recently reported. Chen H et al. (17, 18) proved that a radiomics signature based on MRI was useful for predicting the response to neoadjuvant chemotherapy and early relapse. Zhao SL et al. (19) showed that a radiomics signature extracted from diffusion-weighted imaging (DWI)-MRI prior to treatment improved the estimation of osteosarcoma.

T1WI, T2WI, and CE-T1WI are the most commonly used MRI sequences for bone tumor. T1WI can be used to observe anatomical structures, but sometimes it is difficult to distinguish soft tissue masses from muscle tissues. T2WI can accurately determine tumor margins and reveal, to a certain extent, the total lesion cell density, whereas CE-T1WI can reveal lesion vascularity, establish the degree of malignancy, and identify necrosis and solid components of tumor. Current research has shown that multiple MRI sequences, such as DWI, T2WI/FS-T2WI and CE-T1WI images, can enhance tumor information extraction and thereby augment specificity, and such sequences for bone and soft tissue tumor radiomics analysis yielded favorable outcomes (20–23). In addition, several studies revealed that classifiers originating from varying classifier families exhibit differing performances for different forms of tumors (24–28). We used two well-known machine learning classifiers in this study. The first is LR, which is a machine learning stratification algorithm used for the prediction of the class probability of a given categorical dependent variable. The second is SVM, which generates a decision margin between two classes to facilitate label estimation from one or more feature vectors. Seven radiomics models using these three sequences alone and combined were established in our study (29). Differences in tumor vessel morphology affect tumor vascular permeability (30). Increased vessel permeability may accelerate cancer metastasis and spread; in the absence of blood vessels, tumors cannot develop beyond a critical volume or invade other organs (31). Enhancing MRI in neoplasms represents regions of admixed vascularity and necrosis, in which contrast permeability is elevated owing to damaged vascular integrity (32). Among the seven radiomics modes, the LR and SVM classifiers had excellent performance (AUC=0.914 and 0.929) in the model combining T1WI, T2WI and CE-T1WI. The combination of T1WI, T2WI, and CE-T1WI allowed the detection of morphological information and indirectly reflected the permeability of tissue microvessels. Although the SVM classifier in the T1WI, T2WI and CE-T1WI combined model performed the best (AUC = 0.929), no obvious difference was observed between the two classifiers of each radiomics model.

In addition, among all radiomics models, those including the T2WI parameter (T2WI, T2WI+CE-T1WI, T1WI+T2WI, and T1WI+T2WI+CE-T1WI) exhibited excellent performance (AUC=0.829-0.929). We found that all of the final features that included the T2WI parameter after selection contained ‘GLCM_Correlation’ and ‘firstorder_Mean’ features, which were high-dimensional features that could not be readily interpreted by humans and included comprehensive tumor information. Among these features, the mean, which is a first-order feature, assesses the average grey level intensity within a specified area of interest. The grey level cooccurrence matrix (GLCM) is a second-order feature and is a summary of the frequency of the various combinations of pixel brightness values that occur between neighboring voxels in a given image. GLCM represents the similarity of voxel values along a given direction, whereas homogeneity represents regional grey level uniformity, and correlation establishes the consistency of image texture (33, 34). In prior studies, these features established tumor heterogeneity and were correlated with the histopathological characteristics and prognosis of numerous tumors, such as osteosarcoma, rectal cancer, thymic tumors, and breast cancer (18, 25, 35–37).

Furthermore, we analysed seven discrete radiomics models, two combined models and one clinical model in osteosarcoma patients. The AUC of the clinical model (0.779) was lower than that of the radiomics model. The prediction ability of the combined model was markedly enhanced relative to that of other models, namely, the clinical model and radiomics model, which involved multiple and single sequences. As clinical information may take into account only some aspects of tumors, multiparametric MRI may better reflect of all tumor information (38). Hence, once the clinical and radiomics characteristics were combined, the performance greatly improved. Based on our data, machine learning analysis involving multiparametric MRI radiomics characteristics can accurately and efficiently predict SLM in osteosarcoma.

Our work encountered certain limitations. First, this work was retrospective in nature. Radiomic features are heavily influenced by differences in the acquisition and reconstruction settings. In the present study, although the most commonly used MRI sequences (T1WI, T2WI and CE-T1WI) were selected, MRI image data were acquired from four distinct scanners, which can also influence the acquired characteristics. Device inconsistency within a dataset is a challenging issue, particularly in retrospective analyses. Second, the sample size was quite small, and all the obtained images were collected over several years. We eliminated non-long bone extremity osteosarcoma and patients who did not receive MRI and chest CT prior to surgery, which accounted for the majority of the enrolled patients. In addition, few osteosarcomas simultaneously met both the axial plane and multiparametric MRI requirements. Due to our strict criteria for patient eligibility, it was difficult to gather large datasets. Despite a statistically insufficient sample size, the results of this study may allow for the improvement of future clinical studies with limited sample sizes. A large sample population with multicentre validation is warranted to achieve high-level evidence for future clinical application. Third, we compared only T1WI, T2WI and CE-T1WI sequences of MRI. The most commonly used clinical examination for osteosarcoma is X-ray, and the effectiveness of X-ray radiomics needs to be investigated in future studies. Additional MRI functional data must be included in future evaluations to enhance the accuracy and clinical value of our model.

In conclusion, we established a noninvasive prediction tool involving radiomics and clinical characteristics to predict SLM in osteosarcoma patients. The LR and SVM classifiers exhibited an elevated degree of diagnostic performance while employing a combination of characteristics for distinguishing SLM and lack of SLM in osteosarcoma patients. Among all radiomics models, those including the T2WI parameter exhibited good predictive performance for the prediction of osteosarcoma SLM. The constructed model involving the combination of clinical and radiomics characteristics is more effective in evaluating osteosarcoma SLM relative to the clinical model and radiomics model, and the constructed model can provide a new basis for early clinical intervention in metastasis.

## Data Availability Statement

The original contributions presented in the study are included in the article/supplementary material. Further inquiries can be directed to the corresponding authors.

## Ethics Statement

Written informed consent was not obtained from the individual(s), nor the minor(s)’ legal guardian/next of kin, for the publication of any potentially identifiable images or data included in this article.

## Author Contributions

ZL, JL, XS, and WC contributed to the design and implementation of the concept. ZL and JL contributed equally to this work. JL and ZL contributed in collecting and reviewing patients’ clinical and imaging data. RL and ZL contributed in segmenting the lesions. YL and RL contributed in the statistical analysis of the data. ZL, YL, and RL contributed in building models. All authors contributed to the writing and reviewing of the paper. All authors read and approved the final manuscript.

## Funding

High Level-Hospital Program, Health Commission of Guangdong Province, China (No: HKUSZH201901026).

## Conflict of Interest

Author YL was employed by GE Healthcare China.

The remaining authors declare that the research was conducted in the absence of any commercial or financial relationships that could be construed as a potential conflict of interest.

## Publisher’s Note

All claims expressed in this article are solely those of the authors and do not necessarily represent those of their affiliated organizations, or those of the publisher, the editors and the reviewers. Any product that may be evaluated in this article, or claim that may be made by its manufacturer, is not guaranteed or endorsed by the publisher.
